# Harnessing human tumor organoids for cancer modeling and precision therapy

**DOI:** 10.1093/procel/pwag007

**Published:** 2026-02-16

**Authors:** Tonghai Zhou, Qianyi Wang, Meili Zhang, Yue Huang

**Affiliations:** State Key Laboratory of Common Mechanism Research for Major Diseases, Institute of Basic Medical Sciences & School of Basic Medicine, Chinese Academy of Medical Sciences & Peking Union Medical College, Beijing 100005, China; McKusick-Zhang Center for Genetic Medicine, Department of Medical Genetics, Institute of Basic Medical Sciences & School of Basic Medicine, Chinese Academy of Medical Sciences & Peking Union Medical College, Beijing 100005, China; State Key Laboratory of Common Mechanism Research for Major Diseases, Institute of Basic Medical Sciences & School of Basic Medicine, Chinese Academy of Medical Sciences & Peking Union Medical College, Beijing 100005, China; McKusick-Zhang Center for Genetic Medicine, Department of Medical Genetics, Institute of Basic Medical Sciences & School of Basic Medicine, Chinese Academy of Medical Sciences & Peking Union Medical College, Beijing 100005, China; State Key Laboratory of Common Mechanism Research for Major Diseases, Institute of Basic Medical Sciences & School of Basic Medicine, Chinese Academy of Medical Sciences & Peking Union Medical College, Beijing 100005, China; McKusick-Zhang Center for Genetic Medicine, Department of Medical Genetics, Institute of Basic Medical Sciences & School of Basic Medicine, Chinese Academy of Medical Sciences & Peking Union Medical College, Beijing 100005, China; State Key Laboratory of Common Mechanism Research for Major Diseases, Institute of Basic Medical Sciences & School of Basic Medicine, Chinese Academy of Medical Sciences & Peking Union Medical College, Beijing 100005, China; McKusick-Zhang Center for Genetic Medicine, Department of Medical Genetics, Institute of Basic Medical Sciences & School of Basic Medicine, Chinese Academy of Medical Sciences & Peking Union Medical College, Beijing 100005, China

**Keywords:** tumor organoid, cancer modeling, precision therapy, tumor microenvironment, PDO

## Abstract

Human tumor organoids represent a paradigm shift in cancer modeling, overcoming critical limitations of conventional systems by faithfully recapitulating genetic heterogeneity, three-dimensional architecture, and tumor microenvironment dynamics of patient tumors. Our review explores how human tumor organoids serve as a transformative preclinical platform, bridging the gap between basic research and clinical translations. We highlight recent advances in tumor organoid generation, spanning patient-derived organoids to genetically engineered platforms from normal tissue and human pluripotent stem cells, and their applications in deciphering carcinogenesis, clonal evolution, and metastatic mechanisms. We further examine technological innovations in culture systems that enhance the interpretability and translatability of tumor phenotypes and drug responses. We present an in-depth exploration of how integrated tumor microenvironment co-culture systems—combining immune cells, cancer-associated fibroblasts, and vascular components–enable novel investigations into tumor-stroma-immune crosstalk. Clinically, human tumor organoid biobanks have shown great promise in predicting personalized therapy responses. Emerging technologies like organoids-on-chip platforms, three-dimensionally bioprinting and artificial intelligence-driven analytics are enhancing high-throughput drug screening efficiency and biomarker identification. Despite advances, complete microenvironmental modeling remains challenging, particularly in replicating vascular complexity and systemic immune responses. Future advancements will demand convergence of synthetic biology, functional genomics, and machine learning to transform human tumor organoids from static avatars into dynamic “living biosensors”. In summary, this review provides an in-depth exploration of the organoid field and presents a clear and actionable framework for positioning tumor organoids as indispensable tools in functional precision medicine—a strategy that ultimately bridges mechanistic discoveries with clinical translation.

## Introduction

In recent years, the expanding use of next-generation sequencing (NGS) technologies, coupled with observations of genetic intra-tumor heterogeneity (ITH) in multiple cancer types, has contributed to a progressive shift in research priorities towards precision oncology (Turajlic et al., 2019; Xu et al., 2022). Despite significant progress in novel therapeutic approaches including immunotherapy, cancer remains a leading global health burden, accounting for nearly 10 million annual deaths worldwide, with lifetime incidence rates approaching 20% across both genders (Bray et al., 2024). The disconnect between preclinical models and clinical results represents a major barrier in drug development (Gunjur et al., 2022). This modeling gap directly contributes to the recurrent failure of promising preclinical therapies in human trials.

While genetically engineered mouse models (GEMMs) have yielded many fundamental insights into tumorigenesis, their prolonged development timelines and frequent inability to accurately replicate human-specific pathogenic processes remain significant limitations (Gould et al., 2015). The conventional human cancer models include *in vitro* human two-dimensional (2D) cell cultures and *in vivo* patient-derived xenografts (PDXs) (Drost and Clevers, 2018). However, several limitations have hindered their broader applications. The established cancer cell lines, despite being assumed to be clonal, are in fact highly genetically heterogeneous. They frequently lack matched normal tissue controls, compromising their ability to accurately model tumor-specific biology (Ben-David et al., 2018). Moreover, primary cell cultures grown directly from patient tumors often have a limited lifespan and are slow-growing. Compared to 2D cell cultures, PDX models accurately reflect patient tumor properties (Woo et al., 2021), however they are fundamentally constrained by the immunodeficient murine host, non-physiological microenvironmental interactions, and the inability to faithfully recapitulate metastatic dissemination processes (Dobrolecki et al., 2016; Meehan et al., 2017). Owing to inherent limitations, these models show restricted fidelity in reproducing crucial tumor characteristics, including the tumor microenvironment (TME), molecular evolutionary, heterogeneity, and clinical drug response.

Since the culturing methods for human adult stem cell (ASC)-based organoids were established (Sato et al., 2009), it became feasible to generate human tumor organoids (HTOs) that could recapitulate key structural and functional characteristics of primary tumors. According to the conceptual framework of organoids (Clevers, 2016), HTOs are typically defined as three-dimensionally (3D) cultured, self-assembling microtumor models established from patient-derived tumor tissues separated by mechanical disruption and enzymatic digestion (Drost et al., 2016), a system known as patient-derived organoids (PDOs). Later, methodologies have been expanded to using human stem cell derived-organoids to study the origin of mutational signatures in tumors (Drost et al., 2017; Sachs et al., 2018). Techniques for culturing functional human colon cancer organoids in 3D structures have been championed for more than 10 years (Sato et al., 2011). Shortly after, tumor organoid platforms achieved scalability across multiple cancer types, propelled by the establishment of validated tumor organoid biobanks (Driehuis et al., 2020; Sachs et al., 2018; van de Wetering et al., 2015). In 2015, the first living biobank of patient-derived colorectal cancer (CRC) organoids was established, comprising over 20 clinically annotated specimens. This seminal work experimentally validated the capacity of HTOs to resemble the genomic profiles of their primary tumors (van de Wetering et al., 2015). So far, long-term culture protocols have been established for organoids derived from 10 more tumor types, significantly expanding the repertoire of experimentally tractable tumor models (Ji et al., 2023; Liu et al., 2024; Nakagawa et al., 2025; Wu et al., 2024; Xie et al., 2023). Innovative methodologies also facilitate the integration of PDOs into translational pipelines, empowering both clinical decision-making and high-throughput drug discovery (Cartry et al., 2023; Drost et al., 2017; Pauli et al., 2017; Vlachogiannis et al., 2018). HTOs represent a robust *in vitro* model that recapitulates histopathological and genetic features of primary tumors, maintains cell-cell interactions and differentiation hierarchies, and bridges the gap between bedside observations and bench investigations (Wood and Ewald, 2021; Xu et al., 2022).

## Generation and culture of tumor organoids

### Organoid sources

**Figure 1.**
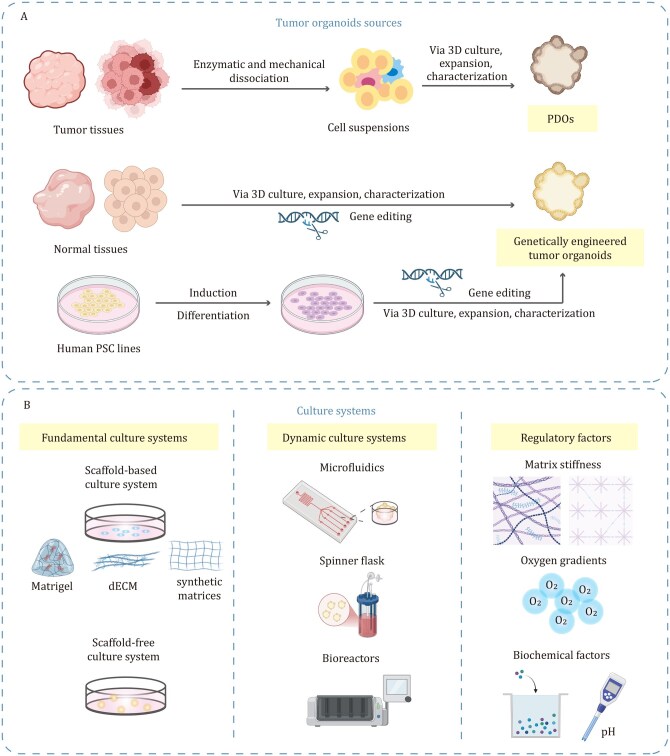
**Integrated overview of tumor organoid sources and culture systems.** (A) Tumor organoids can be derived from tumor tissues (via enzymatic dissociation and 3D culture), normal tissues (with gene editing), or human PSCs (via induction and differentiation, followed by gene editing). (B) Organoid culture methods are illustrated, including (left) fundamental culture systems; (middle) dynamic culture systems; and (right) representative regulatory factors.

The selection of tumor organoid sources directly dictates their application spectrum (**Table 1**). HTOs can be generated through three main routes: (i) PDOs from surgically resected tumor tissue, which are dissociated and cultured in 3D, (ii) tumor organoids generated from normal tissue via genetic modification, and (iii) tumor organoids derived from human pluripotent stem cells (PSCs), which are differentiated and genetically engineered (Artegiani et al., 2020; Hendriks et al., 2021) (Fig. 1A). These sources are widely utilized to explore cancer initiation and progression.

**Table 1.** Comparative overview of tumor organoid sources: advantages, limitations, and applications.

*Organoid Sources*	Advantages	Limitations	Applications	References
***PDOs from surgically resected tumor tissue***	Capture the cellular heterogeneity and spatial organization found within tumors. Strong correlation with clinical drug response. Partially retain elements of the native tumor microenvironment.	Success rate of establishment depends on tumor types. Limited growth potential. Variability in the cellular composition of resected tissues used for organoid generation. Inability to accurately represent the diversity of the cell types in TME. Limited suitability for high-throughput assays. Ethical and logistical constraints in sample acquisition.	Enable the study of tumor behavior and drug response in a patient-specific context. Facilitating the development of tailored therapeutic strategies. Predict clinical outcomes.	(Taurin et al., 2025; Tong et al., 2024; Xu et al., 2024; Zhao et al., 2024)
***Tumor organoids generated from normal tissue via genetic modification***	More readily available than patient tumor samples. Easier to generate in larger quantities, potentially increasing the reproducibility of experiments. Maintain specific tissue traits. Capability of studying early tumorigenesis.	Disability to fully replicate the diversity or behavior of patient-derived tumors. Disability to fully capture the nuances of tumor evolution. Variability between individuals.	Functional studies that can be invaluable for understanding cancer-related processes. Studying somatic mutations and clonal expansion. Personalized cancer therapy.	(Drost et al., 2015; Ganpat et al., 2025; Ishahak et al., 2025; Seino et al., 2018)
***Tumor organoids derived from Human PSCs with gene editing***	Unlimited supply of starting material for organoid generation. Ability to be differentiated into specific cell types. High degree of consistency across experiments. Capability of studying early tumorigenesis.	Lack the complex interactions found in primary tumors. Not fully represent the variety of genetic mutations seen in tumors. Limited tumor heterogeneity. Ethical concerns.	Investigate the role of specific mutations in tumor progression. Generating tumor organoids with specific genetic backgrounds. Create genetically well-defined cancer models. Large-scale drug screening.	(Azar et al., 2021; Heinzelmann et al., 2024; Yan et al., 2023)

PDOs commonly involve enzymatic and mechanical dissociation of fresh tumor tissues to generate cell suspensions enriched with cancer stem cells. PDOs faithfully preserve genetic, epigenetic, and histopathological features of primary tumors, as confirmed by rigorous multi-omics analyses (Lago et al., 2023; Verduin et al., 2023). Whole-genome sequencing, whole-exome sequencing, and targeted panel sequencing have been utilized on PDOs from various cancers, including ovarian, gastric, pancreatic, esophageal, bladder, and CRC (de Witte et al., 2020; Lee et al., 2018; Li et al., 2018; Tiriac et al., 2018; van de Wetering et al., 2015; Zhao et al., 2024). Overall, these data indicate that early-passage PDOs reliably preserve the genomic mutations and copy number alterations present in their original tumors (Nam et al., 2022).

Since cancer often results from the accumulation of mutations in cancer-driver genes (Zhang et al., 2024), it is crucial to understand the mutational processes active in both tissue homeostasis and tumorigenesis. The induced pluripotent stem cell (iPSC)-derived organoids offer an excellent platform for oncology research. They can be generated from patient-derived iPSCs with known oncogenic mutation to study the hereditary tumors, or from the normal donor-derived iPSCs that have been genetically edited to introduce oncogenic mutations and epigenetic modifications (Wang et al., 2024). Although iPSC-derived organoids have demonstrated considerable potential in modeling human development and disease, their direct application in oncology is still emerging, especially in the study of non-hereditary tumors. The concept of using matched iPSC-derived normal organoids as counterparts to tumor organoids has been highlighted as a promising strategy for assessing off-target toxicity and therapeutic safety (Turhan et al., 2021). For instance, patient-specific lung organoids derived from iPSCs, together with lung cancer organoids derived from tumor tissue, have been employed to evaluate cytotoxic effect of cisplatin-encapsulated extracellular vesicles (Kustermann et al., 2024). Nevertheless, this approach remains in an early stage of development, with only limited proof-of-concept studies available, and broader implementation in translational oncology is yet to be achieved.

### Scaffold-based culture systems

Sato and colleagues generated the first ASC-derived organoids from mouse intestinal epithelial stem cells marked by the expression of *Lgr5*. The organoids were grown in Matrigel and maintained in long-term culture (Sato et al., 2009). The 3D tumor models are generally scaffold-based models, where cells grow within engineered matrices that mimic critical extracellular matrix (ECM) properties. These platforms utilize either hydrogel-based or solid polymeric scaffolds to recreate physiologically relevant TME (Fig. 1B) (Unagolla and Jayasuriya, 2022). Among all the natural matrix-based methods, Matrigel is the most extensively used ECM. Commercially available Matrigel predominantly comprises laminin, collagen type IV, and growth factors within a basement membrane extract (BME) matrix, providing the physiological ECM mimicry for 3D cell culture (Kim et al., 2022; Song et al., 2024). However, as Matrigel is extracted from the Engelbreth-Holm-Swarm (EHS) mouse sarcoma, it exhibits inherent batch-to-batch variability in its biochemical composition and physical properties (Aisenbrey and Murphy, 2020; De Vriendt et al., 2023). Another natural matrix, decellularized ECM (dECM), is a biomaterial created by removing cells from a tissue or organ while preserving its native ECM structure (McInnes et al., 2022). dECM is rich in tissue-specific growth factors and signaling molecules, which collectively contribute to shaping a supportive cellular microenvironment. These properties underscore the potential of dECM-based biomaterials to serve as natural, bio-instructive scaffolds that guide tissue-specific cellular functions and promote regenerative processes (Golebiowska et al., 2024). Additionally, synthetic matrices, exemplified by PEG-based systems also play a significant role across various fields of research (Gjorevski et al., 2016). An engineered hyaluronan-gelatin hydrogel system with defined biochemical properties has been developed to support physiologically relevant co-cultures of CRC PDOs and cancer-associated fibroblasts (CAFs), facilitating mechanistic interrogation of TME interactions within a dimensionally controlled setting (Luo et al., 2021).

Studies have demonstrated that stiffness of ECM is critical to the early phase of metastatic spread, encompassing both the movement of cancer cells to secondary locations and their infiltration into the blood and lymphatic vessels (Drost et al., 2015; Lai et al., 2025). A study shown that patient-derived pancreatic ductal adenocarcinoma (PDAC) organoids develop resistance to several clinically relevant chemotherapies when cultured within high-stiffness matrices mechanically matched to *in vivo* tumors (LeSavage et al., 2024). In PDAC organoids, researchers also found that these organoids in stiffer matrices exhibited enhanced cancer stem cell traits and greater resistance to gemcitabine, linked to oxidative stress defenses and drug transporters (Zhao et al., 2025). It has also been found that the drug response is related to the luminal size and pressures of mouse mammary tumor organoids (Fang et al., 2021). The mechanical microenvironment drives the transformation of tumor cell phenotypes, promoting the growth and invasion of colorectal cell spheroids (Nishi et al., 2022). These findings indicate that matrix stiffness and pressure, a critical physical property, can potentially affect the growth and phenotype of tumor organoids cultured in scaffold-based systems.

### Alternative and dynamic culture systems for tumor organoids

Traditionally, long-term organoid culture has been considered dependent on an external matrix. However, emerging evidence suggests that organoid cells may establish their own niche through the secretion of extracellular matrix components (Kozlowski et al., 2021). This insight has spurred the development of matrix-free culture systems (Fig. 1B) for several tissue types, including renal tumor (Seraudie et al., 2023), glioblastoma (Park et al., 2025), melanoma (Angeli et al., 2024), ovarian cancer (Li et al., 2024), and breast cancer (Chang et al., 2024). The commonly used scaffold-free culture methods, such as the hanging drop technique and spinner or rotating flask systems, have already been well summarized in previous studies (Lv et al., 2024). Recent studies have made transformative advances in scaffold-free culture methods, breaking away from conventional approaches. An acoustic virtual 3D scaffold (AV-Scaf) method to achieve 3D tumor organoid culture has been developed, enabling a direct-interacting tumor organoid–immune cell coculture system (Shan et al., 2024).

Both biomechanical and biochemical environments influence cancer cell migration, invasion, and metastasis (Nishi et al., 2022). Hypoxia, a defining feature of solid cancers, has been found to consistently enhanced invasion in patient-derived PDAC organoids (Navarro-Serer et al., 2025) and distinctively modulate the expression of lung cancer and stem cell-associated markers (Li et al., 2025). Despite its significance, convenient methods to simulate *in vivo* hypoxic TME under normoxia conditions remain limited. Recent advances, however, offer promising platforms. For example, a 3D culture system with a core-shell structure (3D-ACS) restricts oxygen diffusion to a certain extent, effectively mimicking hypoxic TME *in vivo* (Ruan et al., 2023). Similarly, 3D bioreactor system can also be used to provide a physiologically relevant environment in terms of oxygen delivery. Researchers have found that oxygen delivery to cultures can be achieved through a polydimethylsiloxane (PDMS) membrane positioned at the bottom of the wells, while microfabricated PDMS pillars further regulate the distribution (Wulftange et al., 2019). These accessible, moderately configured laboratory platforms hold potential for broader application in ­preclinical research (Ruan et al., 2023).

Biochemical factors, such as extracellular pH, are also crucial for predicting and quantifying anticancer drug efficacy (Khan et al., 2024). The acidic microenvironment generated by aberrant glycolysis (the Warburg effect) profoundly influences tumor progression and immune evasion (Kooshan et al., 2024). Incorporation of extracellular pH modulation into organoid culture has enabled the study of acidosis-driven changes in cell signaling, proliferation, and drug response (Richiardone et al., 2025). A method to integrate silica-based fluorescent pH sensors into alginate-based 3D microgel tumor models, combined with computational analysis, is presented (Rizzo et al., 2022). This system enables real-time imaging of multiple cell types in 3D and their pH metabolic interactions, making it a promising platform for drug screening and personalized medicine.

Beyond scaffold-free and biomechanical/biochemical modulation, dynamic culture platforms—notably microfluidic devices and bioreactors—have emerged as powerful tools to further refine tumor organoid culture (Chauhdari et al., 2025). For instance, an innovative vascularized PDOs-on-a-chip with hierarchical, tumor-specific microvasculature is presented, providing a versatile platform to explore tumor-vascular dynamics and anti-vascular drug efficacy (Du et al., 2025). Similarly, a perfused multi-well bioreactor platform was established to assess colorectal cancer liver metastases (CRCLM) organoid response to a chemotherapeutic gradient (Wasson et al., 2023). These dynamic culture platforms provide controlled conditions that support adequate oxygen delivery, efficient exchange of nutrients and metabolites, and balanced cell distribution, thereby maintaining high organoid viability and physiological relevance.

### Cytokine cocktails

Defined cytokine cocktails are indispensable for tumor organoid cultures, simultaneously regulating stemness maintenance, lineage specification, and stromal crosstalk (Zuo et al., 2025). The first intestinal organoid system was supplemented with stem cell niche-regulating factors such as Wnt3a, Noggin, R-spondin 1, and epidermal growth factor (EGF) (Sato et al., 2009). In intestinal crypts, niche-derived signals create a tightly regulated Wnt activity gradient that is highest in the stem cell compartment and progressively decreases along the crypt-villus axis (Walter et al., 2022). Most studies continue to utilize the established combinations with tumor-type specific supplements. The Wnt/β-catenin signaling pathway plays a central role in regulating stem cell homeostasis and maintaining tissue integrity, contributing to both normal and malignant epithelial development and emerging as a promising therapeutic target in cancer treatment (Nusse and Clevers, 2017). In organoid cultures, Noggin functions by binding to BMPs and suppressing BMP-SMADs signaling, which alleviates BMP-mediated inhibition. This action, together with Wnt signaling, promotes stem cell self-renewal (Yuan et al., 2015). EGF induces proliferative signaling cascades that support the self-renewal and expansion of adult stem cell populations within organoids (Abud et al., 2021). We summarize the conventional culture methods of tumor organoids derived from different tissues as well as recent innovative culture approaches in **Table 2**.

**Table 2.** Recent advancements in tumor organoid culture.

Tumor organoid type	Conventional culture methods	Recent innovations in culture systems	Advances in tumor organoid co-culture strategies
***Colorectal cancer organoids***	Matrigel and advanced DMEM/F12 with penicillin/streptomycin, HEPES, GlutaMax, N2, B27, Gastrin, EGF, A83-01, SB202190 and N-Acetylcysteine (Yao et al., 2020).	A tunable designer matrix termed hyaluronan elastin-like protein (HELP) for adult primary tissue-derived, epithelial-only intestinal organoids (Hunt et al., 2021). A growth factor-reduced culture medium containing FGF10, A83-01, SB202190, gastrin, and nicotinamide (Tan et al., 2024).	Direct contact co-culture (Atanasova et al., 2023). Air-liquid interface stratified co-culture (Atanasova et al., 2023). Indirect co-culture (Farin et al., 2023). Microfluidic and 3D bioprinting platforms (Fang et al., 2021).
***Pancreatic cancer organoids***	Growth factor-reduced Matrigel and advanced DMEM/F12 with HEPES, GlutaMax, penicillin/streptomycin, B27, N-Acetylcystenine, Wnt-3a, R-spondin 1, Noggin, EGF, FGF, Nicotinamide and A83-01 (Yan et al., 2019).	A mixed-composition hydrogels: Collagen-Matrigel hydrogels as a better matrix for modeling PDAC biology (Sorzabal-Bellido et al., 2025).	Direct contact organoid-fibroblast co-culture (Schuth et al., 2022). Air-liquid interface stratified co-culture (Tabe et al., 2025). Indirect co-culture (Hwang et al., 2019) Microfluidic and 3D bioprinting platforms (Petrauskas et al., 2025).
***Glioblastoma organoids***	Scaffold-free culture system with DMEM/F12, Neurobasal, GlutaMax, NEAA, penicillin/streptomycin, N2, B27 supplement without vitamin A, 2-mercaptoethanol, and human insulin per well and placed on an orbital shaker rotating (Jacob et al., 2020).	Biomimetic hydrogel platform by integrating a brain-derived dECM with hyaluronic acid methacrylate (HAMA), yielding a composite (1H3D) that closely reflects the ECM characteristics of glioblastoma (GBM) tissue (Zhou et al., 2025).	Direct contact co-culture (Jacob et al., 2020). Indirect co-culture (Sun et al., 2025). Microfluidic and 3D bioprinting platforms (Ning et al., 2022).
***Lung cancer Organoids***	Matrigel and advanced DMEM/F-12 with HEPES, GlutaMax, penicillin/streptomycin, EGF, bFGF, B27, N2, Y-27632, and A83-01 (Shi et al., 2020).	A droplet-based 3D bioprinting strategy based on sodium alginate, hyaluronic acid, and arginine-glycine-aspartic acid peptide is established to construct lung cancer organoid arrays for drug evaluation ( Dong et al., 2023).	Direct contact co-culture (Oh et al., 2025). Air-liquid interface stratified co-culture (Ning et al., 2022). Indirect co-culture (Lee et al., 2024). Microfluidic and 3D bioprinting platforms (Gong et al., 2025).
***Breast cancer organoids***	Basement membrane extract, type 2 and advanced DMEM/F12 with R-Spondin 3, neuregulin 1, FGF 7, FGF 10, EGF, Noggin, A83-01, Y-27632, SB202190, B27, N-Acetylcysteine, Nicotinamide, GlutaMax, HEPES, penicillin/streptomycin, Primocin (Sachs et al., 2018).	A nanofibrillar hydrogel for the initiation and growth of breast cancer PDOs (Prince et al., 2022). Generate and maintain organoids as suspension cultures in reconstituted basement membrane (Matrigel) (Chang et al., 2024).	Direct contact co-culture (Raffo-Romero et al., 2024). Air-liquid interface stratified co-culture (Raffo-Romero et al., 2024). Indirect co-culture (Raffo-Romero et al., 2024). Microfluidic and 3D bioprinting platforms (Maiullari et al., 2025).
***Liver cancer organoids***	Basement membrane extract, type 2 with advanced DMEM/F12 supplemented with penicillin/streptomycin, GlutaMax, HEPES, B27 supplement without vitamin A, N2, N-Acetylcysteine, nicotinamide, recombinant human gastrin I, EGF, FGF10, HGF, forskolin, A83-01, Y27632 and dexamethasone (Broutier et al., 2017).	A biocompatible double-network hydrogel using HAMA, sodium alginate, methacrylamide dopamine (DMA), and c(RGDFC) for liver cancer organoid culture (Zhao et al., 2024).	Direct contact co-culture (Liu et al., 2021). Indirect co-culture (Liu et al., 2021) Microfluidic and 3D bioprinting platforms (Xie et al., 2021).
***Melanoma organoid***	Collagen gel and advanced DMEM/F12 medium with Wnt3a, R-spondin 1, Noggin-conditioned media, inactivated fetal calf serum, HEPES, GlutaMax, Nicotinamide, N-Acetylcysteine, B27 supplement without vitamin A, A83-01, penicillin/streptomycin, Gastrin, SB202190, EGF and FGF-1 (Ou et al., 2023).	/	Direct contact co-culture (Ou et al., 2023). Air-liquid interface stratified co-culture (Cordts et al., 2024). Microfluidic and 3D bioprinting platforms (Bourland et al., 2018).

## Modeling tumor biology with organoids

An increasing number of organoid models have been used to investigate the onset and progression of cancer (Drost and Clevers, 2018). HTOs offer a significant advantage by faithfully replicating the complex features of human tumors, providing a revolutionary platform for advancing our understanding of the fundamental biological principles of cancer (Han et al., 2024). Unlike conventional models, organoids retain the genetic landscape, phenotypic heterogeneity, and 3D structure of primary tumors. Moreover, components of TME can be integrated into co-culture systems, making HTOs an ideal tool for dissecting tumor initiation, progression, heterogeneity evolution, and interactions with the surrounding microenvironment (Wang et al., 2022).

### Modeling carcinogenesis

Tumorigenesis originates from the gradual accumulation of heritable genetic alterations in normal somatic cells. Numerous studies using cellular and animal models have elucidated the key factors and decisive mechanisms involved in the progression from genetic mutations to tumor formation (Baggiolini et al., 2021; Li et al., 2024; Wu et al., 2025; Zhang et al., 2024). However, due to the inability to directly observe or intervene in the earliest phases of tumor development, our understanding of this initial process remains significantly limited. Organoid technology enables the reconstruction of the multistep progression from normal epithelium to precancerous lesions and ultimately to invasive carcinoma under controlled experimental conditions (Kretzschmar, 2021).

Tumor-associated gene mutations have been precisely introduced into normal tissue or cell line-derived organoids using gene editing techniques (**Table 3**). Pioneering work in this field was first established in human CRC. By sequentially engineering key CRC driver genes (*APC*, *KRAS* or *PIK3CA*, *TP53* and *SMAD4*) in healthy intestinal stem cells (ISCs)-derived organoids, researchers successfully modelled CRC progression *in vitro* (Drost et al., 2015; Matano et al., 2015). *TP53*-deficient human gastric organoids exhibited transcriptional and genomic hallmarks of premalignant gastroesophageal lesions despite remaining histologically normal (Karlsson et al., 2023). More recently, CRISPR/Cas9-mediated *ARID1A* knockout (KO) in primary *TP53*^−/−^ human gastric organoids induced morphologic dysplasia, tumorigenicity, and mucinous differentiation (Lo et al., 2021). Beyond gastrointestinal tumors, gene-editing in other healthy organoids induced malignant transformation as well: a study targeting knockout of four breast cancer-associated tumor suppressor genes (*TP53, PTEN, RB1, NF1*) in mammary progenitor cells demonstrated that mutant organoids acquired long-term expansion capacity and formed estrogen receptor-positive luminal tumors upon transplantation into mice (Dekkers et al., 2020). Another study using human induced hepatocytes (hiHeps) from fibroblasts organoids reported that *c-Myc*-induced human hepatocellular carcinomas (HCCs) initiation was associated with alterations in mitochondrion-associated endoplasmic reticulum membranes (MAMs) (Sun et al., 2019). Modeling pancreatic intraepithelial neoplasia was achieved by introducing *KRAS, CDKN2A*, *SMAD4*, and *TP53* mutations into primary human pancreatic duct cells, recapitulating the progression of early pancreatic tumorigenesis (Lee et al., 2017). In human cerebral organoids, tumors can also be induced by introducing CRISPR/Cas9 and sgRNAs in combination with the activated oncogene *HRas^G12V^* and simultaneous disruption of the tumor suppressor *TP53* (Ogawa et al., 2018).

**Table 3.** Genetically engineered HTOs to study tumorigenesis.

Tumor types modeled	Tissue origin	Organoid type	Gene editing method	Genes manipulated	Outcome	References
***Colorectal cancer***	Endoscopic duodenal biopsy samples and normal human colon tissue	Intestinal organoids, colon organoids	CRISPR/Cas9	*APC, KRAS, TP53, SMAD4*	Invasive carcinomas	(Drost et al., 2015)
	Surgically resected intestinal tissues or endoscopic biopsy samples	Intestinal organoids	CRISPR/Cas9	*APC, KRAS, SMAD4, TP53, PIK3CA*	Localized tumors	(Matano et al., 2015)
***Gastric cancer***	Surgical gastric cancer sample from patients	Gastric organoids	siRNA & shRNA	*ARGLU1, TTF2, SSX4*	Activating *ARGLU* and *TTF2* could inhibit tumor growth	(Li et al., 2021)
	Healthy patients undergoing sleeve gastrectomy	Gastric organoids	CRISPR/Cas9	*TP53*	Premalignant gastroesophageal lesions	(Karlsson et al., 2023)
	Normal human gastric corpus samples	Gastric organoids	CRISPR/Cas9	*TP53& ARID1A*	Promotes gastric malignancy	(Lo et al., 2021)
***Pancreatic cancer***	PDAC tissue	PDAC organoids	shRNA	*SIRT5*	Accelerated tumor growth upon transplantation	(Hu et al., 2021)
	Primary human pancreatic duct cells	Purified cells grown in sphere cultures	CRISPR/Cas9	*KRAS, CDKN2A, SMAD4& TP53*	Cells reconstitute stable pancreatic intraepithelial neoplasia structures	(Lee et al., 2017)
***Hepatocellular carcinomas***	hiHeps generated from immortalized umbilical cord fibroblasts	hiHeps organoids	CRISPR/Cas9	*c-Myc*	Bona fide HCCs formation	(Sun et al., 2019)
***Breast Cancer***	Human breast tissue	Breast epithelial organoids	CRISPR/Cas9	*P53, PTEN, RB1, NF1*	Long-term expansion in culture and formed luminal tumors upon transplantation into immunodeficient mice	(Dekkers et al., 2020)
***Glioblastoma***	Embryonic stem cell (ESC) line H9	Human cerebral organoids	CRISPR/Cas9	*RAS&TP53*	Become invasive and destroy surrounding organoid structures	(Ogawa et al., 2018)
***Ovarian cancer***	iPSCs with germline pathogenic *BRCA1* mutations	BRCA1^mut^ fallopian tube organoids	/	*BRCA1*	Increased production of cancer-specific proteins	(Yucer et al., 2021)
***Papillary renal cell carcinoma***	Bone marrow mononuclear cells	Kidney organoids	/	*c-Met*	Induced larger tumors in mice and expressed kidney cancer markers	(Hwang et al., 2019)

PDOs also play an important role in investigating the initiation and progression of tumorigenesis. *APC*-KO Barrett’s esophagus (BE) organoids showed characteristic goblet cell differentiation, recapitulating the critical roles of aberrant Wnt/β-catenin signaling activation in neoplastic transformation of Barrett’s esophagus (Liu et al., 2018). Additionally, RNA interference-mediated gene silencing revealed critical roles of PDOs in tumorigenesis. Knockdown of *SIRT5* using short-hairpin RNAs (shRNAs) increased human PDAC organoid growth and accelerated tumor growth upon implantation (Hu et al., 2021). A study also found that *ARGLU1*-induction and *TTF2*-induction with small activating RNAs (saRNAs) or *SSX4* knock-down with shRNAs could significantly inhibit tumor growth in gastric cancer PDO models (Li et al., 2021).

HTOs derived from patient-specific iPSCs serve as powerful tools for modeling hereditary tumors and investigating their underlying pathogenic mechanisms. Using iPSCs derived from ovarian cancer patients with germline pathogenic *BRCA1* mutations, *BRCA1^mut^* organoids showed an increased production of cancer-specific proteins and survival following transplantation into mice (Yucer et al., 2021). iPSCs-derived embryoid bodies carrying *c-MET* mutations also recapitulated early molecular features of hereditary papillary renal cell carcinoma (PRCC), providing a human model for studying disease initiation (Hwang et al., 2019). These models enable the study of “From normal to malignant progression” to monitor early oncogenic events in real-time at both molecular and cellular levels, identify critical driving factors and susceptible targets, and provide a platform for testing chemoprevention strategies.

### Analysis of tumor heterogeneity and dynamic evolution

HTOs can preserve and recapitulate tumor heterogeneity and clonal evolution, thereby facilitating the study of tumor progression, metastasis, and mechanisms of drug resistance. Tumor heterogeneity, referring to the genetic and phenotypic diversity both within and between tumors, poses a major challenge in cancer research and therapy (Cyll et al., 2017). Organoid evolution models complemented with integrated single-cell sequencing technology provide a powerful platform to characterize tumor heterogeneity as well as tumor evolution. Coupled with spatial transcriptomic analyses, studies confirm that PDOs faithfully recapitulate the cellular diversity of primary tumors, encompassing distinct cancer subclones with unique mutational profiles, stem-like populations, heterogeneous differentiation states, and non-malignant stromal components in co-culture systems (Kratz et al., 2025; Landon-Brace et al., 2023). Patient-derived glioblastoma organoids (PGOs) model tumor heterogeneity by preserving polyclonal subpopulations and key chromosomal alterations such as chromosome 7 amplification and chromosome 10 deletion. Multiplex immunohistochemistry revealed four distinct GBM cell states in PGOs, closely matching the cellular distribution in the primary tumors (Verduin et al., 2023). Similarly, in pancreatobiliary cancers, single-cell transcriptomic and genomic analyses of paired primary tumors and PDOs from cholangiocarcinoma (CCA) and PDAC patients have shown that organoids retain the copy number variation patterns of the parental tumors, while single-cell whole-genome sequencing of PDAC organoids further demonstrates persistent intra-organoid genomic heterogeneity (Krieger et al., 2021; Usman et al., 2022).

HTOs not only capture the genetic and phenotypic heterogeneity of tumors but also enable dynamic tracking of clonal evolution. When combined with single-cell and lineage-tracing technologies, they reveal subclonal transitions, shifts in stemness, and adaptive processes during tumor progression and metastasis, providing critical insights into the mechanisms of malignancy and therapeutic resistance. Delineating the clonal evolution trajectory is pivotal to the understanding of tumor biology. Clonal tracking techniques such as lineage tracing and cellular barcoding have been employed to study the dynamic evolution in tumor. Using viral lineage barcodes, a parallel evolution experiment recapitulating tumor progression in colon cancer organoids has characterized the sequence of chromosomal aberrations and pinpointed recurrent genomic events across distinct subclones (Kester et al., 2022). HTOs derived from primary and metastatic sites offer a valuable platform for comparing tumor progression, enabling direct investigation of clonal evolution and adaptation during metastasis (MacDonald et al., 2025). The progression of CRC has been modeled using paired PDOs from primary and metastatic lesions (Li et al., 2020). Integrated *in vitro*, *in vivo*, and transcriptomic analyses of these paired organoids identified key genes ­associated with CRC liver metastasis (e.g., *SOX2*). Metastasis-derived organoids exhibit significantly enhanced tumorigenic and metastatic potential compared to primary tumor-derived counterparts, highlighting their translational value as prognostic biomarkers for therapeutic stratification (Zheng et al., 2024). Comparative transcriptomic analysis of cancer stem cells from primary CRC PDOs and liver metastasis-derived PDOs has shown that stem/transit-amplifying (TA)-like cancer cells in liver metastases have greater self-renewal capacity than those in primary tumors. Cell trajectory analysis indicated a differentiation trend of stem-like cells toward mature states in primary CRC PDOs, while liver metastasis PDOs exhibited a reverse transition from mature-like to stem-like phenotypes (Li et al., 2020). These findings highlight the elevated self-renewal potential of stem-like cells in metastatic CRC (Mo et al., 2022).

### Reconstruction of tumor organoid–TME interactions

HTOs often lack the full complexity of the *in vivo* TME, which comprises both cellular components such as immune cells, fibroblasts, and endothelial cells, as well as non-cellular elements including the ECM and associated biophysical cues (Crouigneau et al., 2024). Interactions between organoids and these TME constituents are crucial for accurately modeling tumor behavior, progression, and therapeutic response. To bridge this gap, recent research has focused on developing co-culture systems that integrate stromal, vascular, and immune cells to restore cell-cell communication, while also reconstructing the non-cellular TME through tissue-specific dECM that recapitulate matrix-derived biochemical and mechanical cues (Wang et al., 2025). Together, these advances have markedly improved the physiological relevance and translational potential of tumor organoid models.

#### Co-culture with stromal cells

Stromal cells, including CAFs, endothelial cells and mesenchymal stem cells (MSCs), contribute to tumor progression by regulating extracellular matrix, angiogenesis, and metabolism (Zhao et al., 2023). CAFs are a subtype of fibroblasts present in TME. As the main component of stromal cells, they are not themselves malignant cells, but play a crucial role in tumor growth, promoting invasion, and facilitating metastasis (Guo and Xu, 2024). A patient-derived CRC organoid co-culture model with fibroblasts has been developed to explore tumor heterogeneity and interactions within the TME. Unlike traditional organoid models that consist solely of epithelial tumor cells, this system integrates patient-matched CAFs and normal fibroblasts (NFs) to replicate key stromal-epithelial interactions. CAFs and NFs maintained distinct protein expression profiles related to extracellular matrix remodeling, cell migration, and immune regulation. Organoids co-cultured with fibroblasts more closely resembled the morphology and marker expression of primary tumors, whereas organoids cultured alone appeared more uniform (Atanasova et al., 2023).

By incorporating endothelial cells into organoid cultures, vascularized tumor organoids have emerged as advanced models that build upon conventional tumor organoids through the formation of functional vascular networks primarily composed of endothelial cells (Zhou et al., 2025). The incorporation of vasculature enables more physiologic delivery of oxygen and nutrients, as well as efficient removal of metabolic waste (Vila Cuenca et al., 2025). This substantially improves the internal microenvironment, reduces central necrosis, and enhances both viability and long-term stability of the models. More importantly, vascularized systems reproduce the complex interactions between tumor cells and endothelial cells, which are fundamental to tumor growth, invasion and metastasis (Lugano et al., 2020).

Co-culture of tumor organoids with MSCs has emerged as an effective approach to reconstruct the stromal microenvironment. In early studies, host-liver colorectal tumor organoids were generated by embedding CRC spheroids with human MSCs in a liver-mimetic matrix. MSCs supported organoid growth, promoted tissue-like organization, and conferred increased resistance to chemotherapeutic agents compared with monoculture organoids (Devarasetty et al., 2017). More recently, MSCs have been shown to rescue organoid-forming capacity in patient-derived lung cancer samples that otherwise failed to generate organoids, with Kindlin-2–dependent signaling in MSCs driving enhanced proliferation and survival of lung cancer organoids (Sui et al., 2025). In HCCs, an MSC-based organoids-on-a-chip platform was developed in which MSCs form a stromal bed that supports tumor organoid growth and enables functional assessment of patient-specific responses to immune checkpoint inhibitors and targeted agents (Zou et al., 2023). Collectively, these studies illustrate how MSCs co-culture enhance organoid establishment, reinforce tumor–stroma crosstalk, and reveal MSC-mediate mechanisms of stemness, invasion, and therapy resistance across multiple cancer types.

Beyond single-component stromal models, more complex systems integrating multiple stromal cell types have been developed to better mimic tumor heterogeneity and functional interactions within the TME. For example, a patient-derived gastric cancer assembloid (PDGCA) system incorporates patient-matched CAFs, MSCs, and endothelial cells, each expanded in subtype-specific media, to recapitulate critical stromal–epithelial crosstalk. These stromal subpopulations maintained distinct gene expression profiles related to ECM organization, inflammatory signaling, and metabolic reprogramming (Shapira-Netanelov et al., 2025). Assembloids co-cultured with autologous stromal cells more closely resembled the original tumors in cellular architecture, biomarker expression, and transcriptomic signatures, whereas organoids alone exhibited reduced structural complexity and stromal-specific gene expression (Zhao et al., 2023).

#### Incorporating immune cells into tumor organoids

The integration of immune components into tumor organoid systems enables the modeling of tumor–immune interactions and provides a powerful tool for evaluating immunotherapies (Wang et al., 2025). A fused pancreatic cancer organoid (FPCO) model was established by co-culturing patient-derived cancer cells with human iPSC-derived cells, followed by the addition of THP-1-derived macrophages to generate M0-FPCOs. Within this system, macrophages acquired pro-angiogenic properties and facilitated endothelial network formation. Single-cell RNA sequencing revealed five tumor-associated macrophage (TAM) subpopulations, including pro-tumorigenic SPP1-TAMs. These TAMs notably enhanced FPCO survival and promoted tumor cell proliferation (Tabe et al., 2025). Co-culture systems that integrate HTOs and immune cells represent a cutting-edge platform for dynamically modeling tumor–immune interactions. These systems offer a unique opportunity to directly observe how immune cells regulate tumor growth and malignant progression (Porter et al., 2020). T lymphocytes are central effectors of antitumor immunity, capable of specifically recognizing and eliminating malignant cells. As the major adaptive immune component in the TME, tumor-infiltrating T cells critically shape tumor progression and therapeutic response, making their incorporation into tumor organoid co-culture systems essential for modeling immune–tumor interactions (Verma et al., 2022). An autologous immune cell–tumor organoid co-culture system was developed by combining patient-derived peripheral blood lymphocytes with matched HTOs, successfully inducing tumor-specific T cells. These functional tumor-reactive T cells were generated in 50% of patients with mismatch repair-deficient (dMMR) CRC and 33% of those with non-small cell lung cancer, with major histocompatibility complex (MHC) restriction assays confirming the specificity of tumor cell targeting (Dijkstra et al., 2018). Since then, co-culture models combining various cancer organoids with immune T cells have been established, providing a unique platform to study tumor immune responses and predict immunotherapy outcomes (Holokai et al., 2020; Wang et al., 2024). Other TME components, such as tumor-infiltrating lymphocytes (TILs) (Shin et al., 2021), natural killer (NK) cells (Chan and Ewald, 2022), TAMs (Zou et al., 2023), and dendritic cells (DCs) (Lück et al., 2025; Subtil et al., 2023), have also been integrated into organoid co-culture models. These advances enable researchers to investigate how these components interact with cancer cells and influence tumor behavior.

#### Reconstitution of extracellular matrix

The TME comprises both cellular and non-cellular components that collectively shape tumor behavior, progression, and therapeutic response. Building upon the previously discussed culture systems that replicate biochemical and biophysical cues, recent advances have focused on reconstructing the non-cellular TME through dECM-based platforms. These systems provide tissue-specific structural and compositional fidelity, thereby enhancing the physiological relevance of tumor organoid cultures (Varinelli et al., 2025).

An innovative human dECM platform faithfully recapitulates cholangiocarcinoma pathophysiology, preserving native cellular migration patterns, patient-specific molecular signatures, and clinically observed chemoresistance mechanisms. HCCs-derived dECM platforms preserve native transcriptomic and proteomic profiles along with patient-specific chemoresistance mechanisms (van Tienderen et al., 2023). In another study, dECM isolated from the peritoneal cavity has been demonstrated exceptional utility in supporting organoid culture derived from peritoneal metastases, faithfully reproducing critical features of the metastatic niche. Researchers generated dECM scaffolds from both tumor and normal peritoneal tissues of CRC patients with peritoneal metastases, finding that tumor-derived dECM exhibited increased stiffness and distinct structural organization compared to normal dECM (Varinelli et al., 2023). Collectively, these studies demonstrate that tumor-specific dECM scaffolds preserve the molecular identity and biophysical properties of the original TME, providing a promising platform for precision oncology research.

## Translational applications in cancer therapy

HTOs have become essential tools in cancer research and precision medicine, demonstrating tremendous translational potential in drug screening, personalized therapy prediction, and biomarker discovery in recent years (Avci et al., 2024).

### Tumor organoid biobanks for drug screening and precision therapy

Drug screening is a core component of cancer research and therapeutic development. Traditional 2D cell lines often fail to accurately reflect patient-specific heterogeneity due to genetic drift and lack of tumor heterogeneity, and they cannot recapitulate the true TME, making clinical drug efficacy prediction challenging (Huang et al., 2024).

PDOs retain the genetic, molecular, and phenotypic features of primary tumors along with a reconstituted TME, making them ideal platforms for precision drug screening. Tumor organoid biobanks serve as specialized repositories for the systematic collection, preservation, and management of HTOs, providing standardized quality control that greatly enhances their utility in clinical translation and large-scale implementation (**Table 4**) (Xie et al., 2023). In lung cancer research, investigators have employed a mechanical, enzyme-free dissociation technique which preserves the integrity of the native TME to isolate tumor cell clusters from patient-derived lung cancer tissues. Using this approach, they established a biobank comprising 171 personalized patient-derived lung cancer organoids from seven individuals. This organoid biobank enables simultaneous dissection of local immune microenvironment heterogeneity, prediction of immune checkpoint inhibitor responses, and identification of novel immunoregulatory targets such as CD99, thereby providing a mechanistic and modeling foundation for precision immunotherapy in lung cancer (Liu et al., 2024). In gastric cancer research, a PDO biobank of 57 organoids derived from 73 patients was established, with models faithfully preserving the histology of their corresponding tumors and recapitulating patient-specific responses to chemotherapeutics. Drug sensitivity profiling revealed distinct expression signatures, with tumor suppressor genes such as *MSMB* and *TP53INP1* upregulated in 5-fluorouracil (5-FU)- or oxaliplatin-sensitive organoids, whereas chemo-resistant organoids were enriched for *FKBP10*, *L1CAM*, and genes within the WNT/PI3K signaling pathway (Zhao et al., 2024). Similarly, in breast cancer, tumor samples from 75 patients were used to establish 60 PDOs, forming a breast cancer organoid biobank. Histological and transcriptomic analyses showed high concordance between PDOs and matched tumors, and drug sensitivity assays revealed that PDOs responses to agents such as paclitaxel and cyclophosphamide varied markedly between patients yet closely mirrored clinical outcomes, underscoring the translational value of PDO-based drug testing for personalized therapy (Wu et al., 2024). In CRC research, researchers have developed a biobank comprising PDOs from 23 patients with metastatic CRC (mCRC), successfully preserving key molecular features including *RAS/BRAF* mutational profiles as well as tissue architecture. This organoid repository enables accurate prediction of chemotherapy responses, recapitulates clinical heterogeneity, and provides guidance for personalized therapeutic strategies. For example, PDOs sensitivity to oxaliplatin was significantly correlated with tumor regression in corresponding patients, and combination treatment with 5-FU and oxaliplatin at a ratio of 1.8:1 was shown to enhance chemotherapeutic efficacy (Smabers et al., 2024). In another study, grouped-seq technology integrates superhydrophobic micropillar array chips (SMARchip) with cost-effective barcode synthesis to enable high-throughput combined phenotypic and transcriptomic drug screening in CRC PDOs. This approach preserves tumor heterogeneity while providing insights into drug mechanisms of action (Wu et al., 2022). In brain tumor research, individualized patient tumor organoids (IPTOs) are generated by engrafting patient-derived tumor tissues into iPSC-derived mini-brain organoid capsules, yielding a biobank of 326 organoids spanning 48 tumor types. This platform preserves the molecular features and intra-tumoral heterogeneity of the original tumors and enables *ex vivo* modeling of entities that are traditionally difficult to culture. Moreover, it has shown high accuracy in predicting glioblastoma responses to temozolomide, as well as broader patient-specific responses to chemotherapy, targeted therapy, immunotherapy, and cell-based therapies across hospitals (Peng et al., 2025).

**Table 4.** Establishment and application of different tumor organoid biobanks.

Cancer type	Patients/PDOs	Establishment method	TME components	Applications	Reference
***Lung Cancer***	7 patients/171 PDOs	*Mechanical mincing* Tissue mincing → 40–100 μm filtration → Matrigel embedding → MoSMAR-chip culture	T cells, macrophages, CAFs	• Anti-PD-1 response evaluation • Tumor-reactive T cell identification • CD99-mediated T cell synergy	(Liu et al., 2024)
***Breast Cancer***	75 patients/60 PDOs	*Enzymatic digestion* Tissue dissection → Collagenase II → 100 μm filtration → BME embedding	T cells, macrophages, endothelial cells	• 22-drug IC50 screening • Personalized therapy guidance • Clinical response prediction (62.5% match)	(Wu et al., 2024)
***Colorectal Cancer***	23 patients/23 PDOs	*Dual-enzyme digestion* Collagenase IV + Dispase I → 100/20 μm filtration → Matrigel embedding	T cells, macrophages	• 5-step optimized drug screen • Clinical response prediction	(Smabers et al., 2024)
***Glioblastoma***	35 patients/171 PDOs	*Host-organoid embedding* Tissue fragments → iPSC-derived brain organoids → Suspension culture	T cells, B cells, myeloid cells, endothelial cells, mast cells	• Temozolomide response prediction • Resistance mechanism (scRNA-seq)	(Peng et al., 2025)
***Esophageal Squamous cell carcinoma***	40 patients/24 PDOs	*Enzymatic + mechanical* Tissue mincing → Dispase I + trypsin → 70 μm filtration → Matrigel embedding	Macrophages, T cells, endothelial cells	•Chemoresistance mechanism (NRF2 activation) • Biomarker discovery (*ALDH3A1/SPP1*) • Drug screening	(Nakagawa et al., 2025)
***Gallbladder Cancer***	41 patients/5 PDOs	*Collagenase IV digestion* Tissue dissociation → 100 μm filtration → Matrigel embedding	T cells, B cells, macrophages, endothelial cells	• Dual-target inhibitor screen (CUDC-907) • Prognostic biomarkers (*HDAC1/2/6*) • scRNA-seq heterogeneity	(Yuan et al., 2022)
***Gastric Cancer***	73 patients/57 PDOs	*Collagenase IV + Dispase I* *digestion* Tissue mincing → Dual-enzyme digestion → 70 μm filtration → Matrigel	T cells, CAFs	• Chemosensitivity prediction • Identification of gene markers • CAF co-culture resistance model	(Zhao et al., 2024)
***Liver Cancer***	57 patients/65 PDOs	*Collagenase IV digestion* Tissue dissociation → 100 μm filtration → Matrigel embedding	T cells, macrophages	• Metabolic subtyping • Discovery of metabolic targets • Lenvatinib + Temsirolimus synergy	(Ji et al., 2023)
***Thyroid Cancer***	8 patients/10 PDOs	*Collagenase II digestion* Tissue mincing → Collagenase II → 70 μm filtration → Matrigel	CAFs	• BRAF-targeted therapy validation • ERα-mediated proliferation • Spatial heterogeneity modeling	(Chen et al., 2021)

The establishment of organoid biobanks for a broadening spectrum of tumor types has now extended to include rare cancers—such as pseudomyxoma peritonei (Martínez-Quintanilla et al., 2024), rhabdomyosarcoma (Savary et al., 2023), and neuroblastoma (Fusco et al., 2019). These rare tumor organoid biobanks offer much-needed, physiologically relevant models for clinical research, representing a major step forward in understanding and treating rare malignancies. PDO models accurately recapitulate the patient-specific complexity of tumors *in vitro*, enhancing the efficiency and precision of selecting the most suitable drugs for individual patients (Nie et al., 2021).

### Identification and validation of predictive biomarkers

Predictive biomarkers, as a core element of precision medicine, play a crucial role in optimizing clinical treatment decisions by prospectively assessing patients’ sensitivity to specific therapies (Chan et al., 2024). This enables the avoidance of ineffective treatments and significantly reduces the risk of drug-related toxicities. However, traditional biomarker development approaches, such as genomic sequencing and proteomics, have notable limitations in functional validation. Although these technologies systematically characterize the molecular features of tumors, they cannot directly capture the dynamic biological responses to therapeutic interventions (Zhou et al., 2024). PDOs therefore provide a complementary, functionally relevant platform for discovering and validating predictive biomarkers.

Patient-derived gastric cancer organoids were used to functionally demonstrate that selective *STAT3* inhibition with *W1131* induces ferroptotic cell death and helps overcome chemoresistance, consistent with effects observed in xenograft and patient-derived xenograft models. These PDO-based data highlight the *STAT3*–ferroptosis axis as a therapeutically actionable vulnerability in advanced gastric cancer (Ouyang et al., 2022). In ovarian cancer, PDOs from *BRCA1*-deficient and PARPi-resistant tumors were used to functionally profile *USP1* inhibitors. Correlations between *ex vivo* drug responses and molecular readouts revealed that accumulation of single-stranded DNA breaks, elevated *RAD18*, and high *USP1* mRNA expression—both at baseline and after PARPi exposure—serve as predictive biomarkers for USP1i sensitivity and response to USP1i–PARPi combination therapy, thereby supporting patient stratification and pharmacodynamic monitoring (Somuncu et al., 2025). Bladder cancer organoids derived from patient tumors were used as an *ex vivo* platform to test cisplatin in combination with the *NAT10* inhibitor remodelin. In these models, remodelin markedly enhanced cisplatin-induced apoptosis and reduced proliferation, functionally linking *NAT10*-driven ac4C RNA modification to cisplatin resistance and supporting *NAT10* and its downstream ac4C–AHNAK axis as predictive biomarkers and therapeutic targets for cisplatin-based therapy in bladder cancer (Xie et al., 2023). By integrating 3D organoid models with network-enhanced machine learning algorithms, a novel biomarker discovery framework was developed that successfully predicted drug responses in CRC (5-FU) and bladder cancer (cisplatin) patients. IC50 data from patient-derived CRC organoids and bladder cancer organoids demonstrated strong concordance with clinical outcomes. This approach has since been expanded to biomarker mining for 44 anticancer drugs, offering a powerful tool to bridge the “*in vitro*-to-clinical translational gap” and advance cancer therapy toward algorithm-driven precision medicine (Kong et al., 2020).

In the future, combining HTOs with innovative technologies is expected to accelerate the translation of biomarkers into clinically actionable tools, paving the way for truly personalized cancer therapy (Kim et al., 2025).

### Patient-specific therapy prediction and clinical correlation

**Figure 2.**
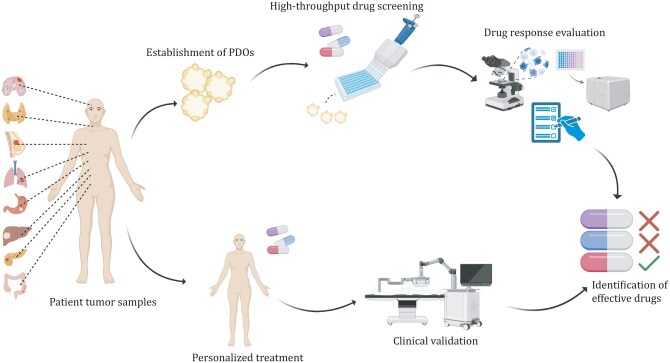
**Schematic workflow of PDO-based personalized drug screening and treatment.** Tumor tissues from various organs (e.g., brain, lung, liver, colon) are collected from the patient to generate PDOs. These organoids are then subjected to drug screening *in vitro* to evaluate the efficacy of various chemotherapeutic agents. The drug response of PDOs correlates with the clinical response observed in the patient, highlighting the potential of PDO-based platforms for guiding personalized cancer therapy.

Drug screening platforms have been effectively implemented using PDOs, which faithfully replicate tumor heterogeneity and the complex TME by capturing patient-specific *in vivo* characteristics (Thorel et al., 2024). Building on this advantage, PDOs allow for rapid and precise *in vitro* assessment of drug sensitivity in individual tumors, thereby supporting the development of personalized therapeutic strategies tailored to each patient (Tong et al., 2024) (Fig. 2).

The ability of PDOs to accurately predict patient-specific drug responses is central to their clinical value. Indeed, numerous studies have demonstrated a strong concordance between PDO drug responses and actual clinical outcomes (Zhao et al., 2024). For instance, a PDO platform derived from seven patients with high-grade serous ovarian cancer (HGSOC) revealed that organoid responses to carboplatin/paclitaxel combination therapy significantly correlated with clinical responses. Notably, effective treatment regimens were identified for 88% of the patients, with combination therapies of carboplatin and gemcitabine successfully overcoming resistance to monotherapies. This study highlighted the value of PDOs in predicting individualized treatment outcomes and capturing tumor heterogeneity (de Witte et al., 2020). Similarly, a PDOs platform was developed for locally advanced rectal cancer (LARC), demonstrating that patients whose PDOs were sensitive to 5-FU exhibited significantly better clinical responses than those with resistant PDOs. This further confirmed the predictive potential of organoid-based drug sensitivity testing for patient prognosis (Lv et al., 2023). As the value of HTOs in personalized drug screening continues to be explored, an increasing number of clinical studies are attempting to integrate organoid-based drug screening into patient treatment workflows. High-throughput screening of patient-derived tumor organoids can serve as an auxiliary tool to help select optimal therapies for cancer patients (Martini et al., 2023).

In clinical practice, PDOs are expanded from tumor tissues and screened against panels of chemotherapeutic or targeted agents (Tong et al., 2024). The resulting drug response profiles are then provided to clinicians as decision-support tools to guide individualized treatment strategies. Importantly, this concept has already progressed from preclinical feasibility to the clinical trial stage (Chen et al., 2021). Recently, a large number of clinical trials involving PDOs have been recorded globally, including feasibility studies as well as intervention trials aimed at directly evaluating precise treatments based on organ tissue engineering (Blandino et al., 2024; Navarro et al., 2025). For example, in HCCs, a trial is evaluating PDOs that integrate patient-derived microbiota and immune components to predict systemic therapy responses (NCT06929845). Another study is using liver biopsy-derived PDOs to test molecular features and *in vitro* drug efficacy (NCT06355700). In ovarian cancer, the clinical trial is assessing whether high-grade serous ovarian cancer organoids can reliably predict treatment response (NCT04555473). For lung cancer, ongoing studies are comparing organoid drug-response phenotypes with clinical outcomes (NCT03979170; NCT06406608). Similar strategies are being explored across other malignancies, including pancreatic cancer (NCT06666803), breast cancer (NCT05177432), gastric cancer (NCT05842187), and colorectal cancer (NCT05832398). These trials show that organoid-based prediction is evolving from a conceptual framework into a clinically testable approach, aiming to translate *in vitro* screening into actionable treatment decisions and shift oncology from population-based regimens toward personalized precision medicine (Chen et al., 2021). To provide a comprehensive overview of ongoing translational efforts, we summarize representative clinical trials involving PDOs across multiple cancer types (2020–2025) in **Table 5**. Collectively, these studies highlight the growing integration of organoid-based functional testing into clinical decision-making.

**Table 5.** Ongoing clinical trials involving human tumor organoids (2020–2025).

Cancer type	NCT number	Title	Recruitment status	Interventions		Primary purpose	Organoid application
***Lung cancer***	NCT03979170	PDOs of lung cancer to test drug response	Active/Recruiting		Tumor tissue collection; PDO culture and *ex vivo* drug testing	Predictive correlation	PDO drug-response profiling vs patient outcomes
	NCT04859166	Prospective primary human lung cancer organoids to predict treatment response	Completed		Primary lung cancer-derived organoid culture; drug testing	Predictive	PDO predictive platform for lung cancer
	NCT03655015	Patient-derived organoid model and circulating tumor cells for treatment response of lung cancer	Recruiting		PDO and circulating tumor cells (CTCs) culture; correlative analyses	Correlative research	PDO + CTCs for baseline and response prediction
	NCT06406608	Patient-derived organoid drug sensitivity guided treatment for drug-resistant recurrent NSCLC	Active/Recruiting		PDO-guided selection of treatment for drug-resistant NSCLC	Treatment guidance	PDO-guided therapy selection
***Pancreatic cancer***	NCT05842187	*In Vitro* Organoid drug sensitivity-guided treatment for metastatic pancreatic and gastric cancer	Recruiting		Biopsy/ascites specimen for organoid culture; organoid-guided drug selection	Treatment	PDO drug sensitivity to guide therapy
	NCT06666803	Functional, personalized and integrated profiling of biopsied pancreatic tumors	Not yet recruiting		Biopsy-derived organoid + multi-omics profiling	Feasibility/personalized profiling	PDO-guided profiling and treatment
	NCT05351983	PDOs drug screen in pancreatic cancer	Completed		Surgical biopsy-derived PDOs for drug screening	Feasibility/drug testing	PDO drug screening
***Hepatocellular carcinoma***	NCT06929845	Organoid models of hepatocellular carcinoma	Recruiting		PDO generation; integration with microbiota/immune components	Translational/predictive	HCCs PDOs for therapy prediction and TME studies
	NCT06355700	Hepatocellular carcinoma liver organoids	Recruiting		Organoid establishment from liver biopsy/tissue	Feasibility/characterization	PDO establishment and characterization
***Ovarian cancer***	NCT04555473	Translational analysis in longitudinal series of ovarian cancer patients (organoid-based)	Active/Recruiting		Serial tumor sampling for PDO generation	Observational/biomarker validation	PDO reliability vs patient response
	NCT04768270	The culture of ovarian cancer organoids and drug screening	Active/Recruiting		Tumor specimens from surgery → PDO culture → drug sensitivity testing	Exploratory/precision therapy guidance	Compare organoid drug sensitivity with patient response, validate PDO as precision tool
***Breast cancer***	NCT05177432	Quadratic phenotypic optimization platform (qpop) coupled with pdos for breast cancer	Active/Recruiting		PDO-based *ex vivo* drug combination screening (QPOP)	Treatment selection/optimization	PDO platform to inform combination therapy
	NCT06315868	Breast cancer subtype characterization through patient’s derived organoids	Active/Recruiting		Surgical tumor samples for PDO establishment and profiling	Characterization	PDO subtype characterization and drug testing
***Colorectal cancer***	NCT05832398	Precision chemotherapy based on organoid drug sensitivity for colorectal cancer	Active/Recruiting		PDO generation and drug testing	Precision treatment guidance	PDO-based drug sensitivity testing
	NCT04371198	PDOs for rectal cancer	Registered		Rectal tumor biopsy specimens → PDO establishment → ex vivo assays	Feasibility and correlative validation of PDO establishment and correlation with clinical response	Use PDOs to map drug sensitivity or correlate with therapeutic outcome in rectal cancer
***Esophageal cancer***	NCT03283527	Organoid based response prediction in esophageal cancer	Recruiting		PDO derivation; correlate with neoadjuvant therapy response	Predictive biomarker	PDO prediction of therapy response (esophageal)
***Gastroesophageal cancer***	NCT06332716	Research on the correlation between organoid drug sensitivity testing and precise treatment of gastrointestinal tumors	Recruiting		PDO generation across digestive tumors (esophagus, stomach, colon)	Correlative/predictive	Cross-digestive PDO drug-response correlation
***Pleural malignancies***	NCT06959173	Organoid-guided personalized treatment of pleural malignancies effusion	Active/Recruiting		Pleural tissue-derived PDOs for drug sensitivity testing	Treatment guidance	PDO-based drug sensitivity to guide pleural cancer therapy
***Osteosarcoma***	NCT06064682	An organoid-based functional precision medicine trial in osteosarcoma	Active/Recruiting		PDO drug screening to guide therapy	Treatment selection/precision medicine	PDO-guided therapy in sarcoma
***Hematologic malignancy***	NCT03890614	Novel 3D hematological malignancy organoid to study disease biology and chemosensitivity	Recruiting		Bone marrow aspirate-derived organoids; chemosensitivity assays	Correlative/feasibility	3D hematologic organoids for chemosensitivity testing

In addition to predictive accuracy, the turnaround time for establishing and testing HTOs is a critical determinant of their clinical integration. In most protocols, PDOs can be established within four to six weeks, which is considerably faster than PDXs that often require six to eight months (Bose et al., 2021). Yet, this timeline may still be too long for urgent clinical decision-making, particularly in aggressive malignancies. Studies in CRC have reported organoid building within one to four weeks after seeding, and ovarian cancer organoids can sometimes be established in as little as two to three weeks (Nanki et al., 2020; Tan et al., 2024). Once established, drug testing typically requires less than two weeks (Foo et al., 2022), and in some gastric cancer models viability assays can produce readouts within five to six days (Alzeeb et al., 2022). Recent technological advances are further compressing this timeline. For instance, droplet microfluidic-generated Micro-Organospheres (MOS) can be established and drug-tested within approximately two weeks, with responses correlating closely to patient outcomes (Yang et al., 2025). High-throughput mini-organoid systems have also been reported to initiate culture within a week and achieve predictive accuracies of around 80% (Zuo et al., 2025).

## Emerging technologies and cross-disciplinary integration in tumor organoid research

**Figure 3.**
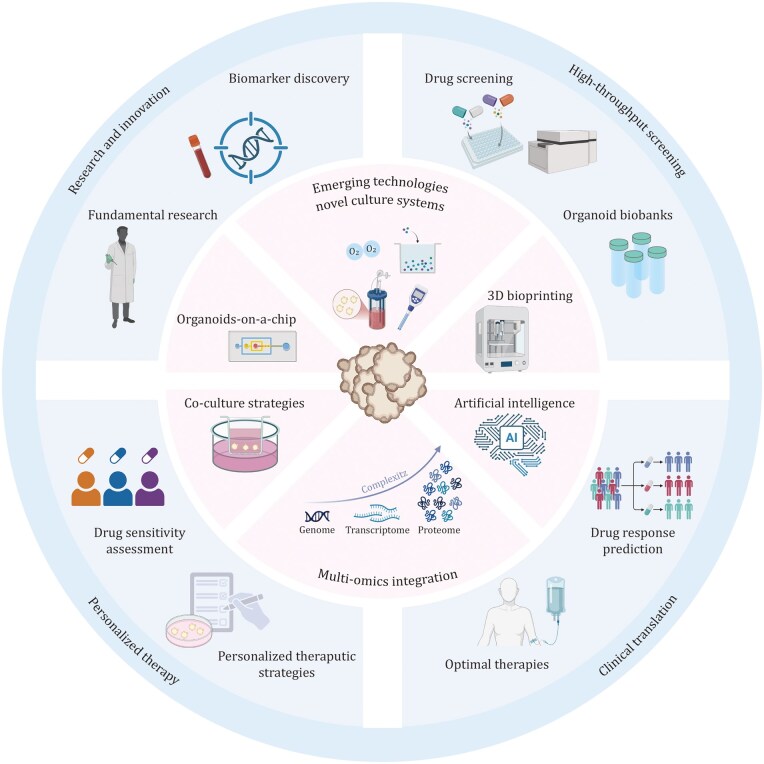
**Integration of emerging technologies and clinical applications of HTOs.** The inner circle highlights emerging technologies, including novel culture systems, 3D bioprinting, artificial intelligence, multi-omics integration, co-culture strategies, organoids-on-a-chip etc. The outer circle highlights key translational applications, including high-throughput screening for rapid experimentation, clinical translation linking research to patient care, personalized therapy focusing on tailored treatment strategies, and research and innovation driving new biomarker discovery. These components collectively bridge fundamental research with clinical outcomes to optimize therapeutic strategies.

Despite their indispensable role in tumor modeling and clinical drug testing, HTOs face several critical challenges that limit broader application. These include poor vascularization, variable reproducibility, architectural heterogeneity, incomplete microenvironment reconstitution, and intra-clonal variability (Wang et al., 2025). These shortcomings underscore the urgent need for advanced platforms that can systematically capture tumor heterogeneity, support vascular integration, and enable long-term dynamic monitoring. Emerging novel technologies, such as novel culture systems, 3D bioprinting, artificial intelligence (AI), multi-omics integration, co-culture strategies and organoids-on-a-chip, are addressing these gaps. Organs-on-a-chip systems recreate physiological gradients and perfusable vasculature, thereby improving nutrient delivery and modeling metastatic dissemination (Vila Cuenca et al., 2025). 3D bioprinting allows precise spatial organization of tumor and stromal components, while synthetic hydrogels such as PEG-based matrices reduce batch variability compared with Matrigel (Wang et al., 2025; Zhao et al., 2025). AI enhances organoid-based drug testing by extracting predictive features from imaging and multi-omics datasets, facilitating individualized therapy prediction (Zhou et al., 2025). In parallel, CRISPR-based gene editing and synthetic biology approaches expand the functional utility of HTOs, enabling mechanistic studies of oncogenic drivers and resistance pathways (Artegiani et al., 2020). These approaches better recapitulate the complex tumor stroma, enable inter-organ and intra-organ communications, permit modeling of multi-organ metastatic processes, and facilitate anti-cancer drug discovery (Fig. 3).

### Organoids-on-a-chip

“Organs-on-a-chip” platforms are microphysiological systems that recreate key structural and functional aspects of human organs with microfluidic devices. By precisely controlling fluid flow, biochemical gradients, and cell-cell or cell-matrix interactions, these systems can simulate organ-level pathophysiology with remarkable fidelity (Ingber, 2022). However, current tumor-on-a-chip models derived from conventional cell lines often fail to capture the full heterogeneity of human tumors. This limitation has driven the recent convergence of microfluidic technology with patient-derived organoid culture, giving rise to innovative “organoids-on-a-chip” systems that better recapitulate native tissue microenvironments (Zhao et al., 2024). Compared to traditional organoid cultures that rely on passive nutrient diffusion through media, vascularized tumor organoids-on-a-chip systems incorporate functional, perfusable microvascular networks (Shirure et al., 2018). This approach has proven particularly valuable for studying complex tumor-vascular interactions, metastasis heterogeneity, and patient-specific drug responses (Du et al., 2025). This system also serves as powerful tools for evaluation of drug efficacy. Patient-derived CRC organoids cultured in microfluidic devices showed expected sensitivity to first-line 5-FU treatment, validating the system’s reliability for drug screening applications (Pinho et al., 2021). Notably, these systems can dramatically reduce the time required for drug sensitivity testing compared to conventional organoid culture (Shirure et al., 2018). The integration of superhydrophobic microwell array chip (InSMAR-chip) with lung cancer organoids has further accelerated this process, allowing patient-specific drug response prediction within one week (Hu et al., 2021). These systems have been used to evaluate drug efficacy against both tumor cells and human umbilical vein endothelial cells (HUEVCs), demonstrating their value for personalized treatment development (Bang et al., 2025). Parallel progress has been made with esophageal adenocarcinoma models, where stroma-inclusive microfluidic platforms successfully predict responses to neoadjuvant chemotherapy by faithfully recreating tumor-stroma interfaces (Pal et al., 2025). These platforms also provide unprecedented opportunities to study metastatic processes. By culturing intestinal tumor organoids adjacent to microvascular networks, researchers have successfully visualized the release of CTCs clusters into vascular channels, enabling identification of key metastatic drivers (Ikeda et al., 2025). An innovative vascularized PDOs-on-a-chip platform was developed, enabling comprehensive investigation of tumor-vascular interactions. Importantly, their system revealed significant correlations between PDOs’ angiogenic potential and migratory capacity with clinical metastatic outcomes, demonstrating its utility for metastasis research and assessment (Du et al., 2025).

### 3D bioprinting

3D bioprinting has emerged as another transformative technology for tumor organoid research. This biofabrication approach allows precise spatial arrangement of cells, bioactive materials, and supporting components to create complex tissue architectures based on computer-aided designs (Moroni et al., 2018). For cancer modeling, bioprinting offers unique advantages in reconstructing the TME through controlled deposition of stroma cells, vascular/lymphatic vessels, and extracellular matrix components. The incorporation of HTOs into bioprinted constructs provides an effective strategy to establish miniaturized tumor units within fully engineered 3D niches (Ning et al., 2022; Nothdurfter et al., 2022). By bioprinting living components directly into prefabricated microfluidic devices in precise spatiotemporal arrangements, researchers can create increasingly sophisticated models that capture hierarchical tissue organization (Meng et al., 2019).

Recent advances have further expanded the utility of bioprinting in organoid research. Multi-material bioprinting enables the simultaneous deposition of diverse cell types and ECM analogs to better mimic stromal heterogeneity, while coaxial printing has been applied to generate perfusable vascular channels that improve nutrient delivery and metastatic modeling (Liu et al., 2025). The use of photocrosslinkable hydrogels and synthetic bioinks provides reproducible mechanical and biochemical properties, thereby overcoming the batch variability associated with Matrigel (Lai et al., 2024). Embedded bioprinting-enabled arrayed PDOs (Eba-PDOs) provide a novel CRC model that faithfully recapitulates key TME features while preserving patient-specific characteristics, enabling more accurate drug response prediction and advancing precision medicine applications (Han et al., 2025). In addition, the integration of bioprinting with organs-on-a-chip systems has yielded vascularized, perfusable tumor models that permit quantitative analysis of drug diffusion and immune cell infiltration, thus bridging the gap between static cultures and physiologically relevant tumor ecosystems (Qu et al., 2021).

Collectively, these developments position 3D bioprinting not only as a fabrication tool but as a versatile platform for precision oncology, enabling high-fidelity modeling of tumor biology and accelerating translational applications.

### High-throughput screening platforms

As organoid technologies and associated biobanks continue to advance, increasingly high-throughput screening methodologies are being developed. By integrating microfluidic systems with multi-omics approaches, researchers have established standardized platforms for organoid-based drug screening that are both cost-effective and information-rich, thereby accelerating the progress of precision oncology (Yang et al., 2025). Notably, a hybrid platform combining bioprinted standardized organoid cultures with high-speed live cells interference imaging (HSLCI) has been developed, enabling high-throughput drug screening at single-organoid resolution. This technology allows for real-time monitoring of the dynamic evolution of drug sensitivity and resistance, while maintaining the intrinsic heterogeneity of the tumor (Tebon et al., 2023). This single-organoid tracking approach can identify sensitive and resistant subpopulations, facilitating the design of combination therapies. Moreover, a single experiment can screen over 900 organoids at a cost lower than traditional PDX models. This drug screening platform is applicable to PDOs and has been used for rapid drug sensitivity testing in neurofibromatosis (Nguyen et al., 2024).

More broadly, high-throughput screening platforms are rapidly advancing the translational potential of HTOs by enabling large-scale, standardized drug testing within clinically relevant time frames. Miniaturized culture formats such as 384- and 1536-well plates have allowed hundreds of patient-derived samples to be tested in parallel, while liquid-handling automation ensures reproducibility and reduces operator bias (Li et al., 2022). Integration with advanced imaging and AI-driven analytics adds another layer of scalability, allowing label-free quantification of cell viability, morphology, and drug-induced phenotypic shifts in real time (Deben et al., 2023). These platforms not only enhance screening throughput but also broaden the range of readouts, from cytotoxicity to pathway-specific signaling responses. Recent innovations include barcoded organoid libraries that permit multiplexed drug testing across hundreds of patient samples, supporting population-level precision medicine studies (Deben et al., 2024).

Together, these high-throughput innovations are transforming organoids from laboratory models into scalable platforms capable of informing both individualized therapy and large-scale drug discovery pipelines (Zuo et al., 2025).

### Artificial intelligence (AI)

Recent years have witnessed the deployment of AI approaches to enhance the predictive power of organoid models. AI algorithms can analyze large datasets to optimize culture protocols, develop automated tools for label-free morphological assessment, and establish correlations between cellular features and genomic profiles (Bai et al., 2024; Qi et al., 2025). Platforms like MOrgAna demonstrate how machine learning can extract meaningful biological insights from complex organoid imaging data (Gritti et al., 2021). AI-powered image analysis tools such as OrBITS and OrganoID have shown remarkable success in high-throughput drug screening using HTOs (Deben et al., 2023; Matthews et al., 2022).

Beyond imaging, deep learning models are increasingly applied to integrate multi-omics layers, linking single-cell transcriptomes, mutational landscapes, and metabolic signatures with organoid phenotypes (Athaya et al., 2023). These approaches enable mechanistic biomarker discovery and stratification of patient subgroups that are not evident from conventional assays. Further validation comes from studies showing AI’s ability to predict anti-cancer drug efficacy in patients based on organoid responses. The SiQ-3D platform enables quantitative analysis of cell-cell interactions in tumor organoid system, providing new insights into tumor biology (Liu et al., 2024). Another important frontier is the development of reinforcement learning frameworks for adaptive therapy design, in which AI continuously updates drug response models based on longitudinal organoid data (Maramraju et al., 2024). This concept transforms organoids into living biosensors that inform real-time treatment decisions. Additionally, explainable AI (XAI) methods are being introduced to reveal which cellular or molecular features drive drug response predictions, increasing trust and interpretability for clinical adoption (Qadri et al., 2025).

These technological synergies are revolutionizing cancer drug discovery by enabling more precise and patient-centered therapeutic predictions. A compelling example of this convergence is SIGX1094R, the first AI-designed drug candidate validated using gastric cancer organoid models. This therapy has received investigational new drug (IND) approvals from both the U.S. FDA and China’s NMPA, demonstrating the clinical translation potential of integrated AI-organoid platforms (NCT06739291). Looking ahead, coupling AI with high-throughput HTO datasets and federated learning across biobanks could accelerate global precision oncology while ensuring data privacy and security (Frascarelli et al., 2023). As these technologies mature, their combined application promises to transform both basic cancer research and clinical practice. The integration of multi-omics data with AI-optimized organoid systems is particularly exciting, offering unprecedented opportunities for personalized medicine. Taken together, these advances position tumor organoid technology as a cornerstone of next-generation cancer research and drug development.

## Challenges and future perspectives

### Toward dynamic patient-specific ecosystems

To create truly patient-specific HTOs, vascular and immune components must be integrated in a dynamic, physiologically relevant manner. Perfusable vasculature should go beyond simple endothelial monolayers. Microfluidic platforms that incorporate pericytes and reproduce physiological shear stress can generate perfused vascular networks, thereby enhancing organoid maturation and function—an approaches that should be standardized for tumor modeling (Quintard et al., 2024). Equally important is immune reconstruction: recent work shows that organoid systems can self-organize tissue-resident immune compartments when supplied with matched hematopoietic elements and appropriate niche cues, indicating a feasible path to generate de novo resident T cell populations within HTO–ECM composites (Recaldin et al., 2024). Looking ahead, future models will likely combine vascular and immune modules in integrated platforms, enabling dynamic crosstalk between endothelial, stromal, and immune cells. Such designs could reproduce processes like metastatic intravasation, immune evasion, and tertiary lymphoid structure formation, which remain poorly captured by current HTO systems.

Rapid, clinically actionable workflows are also essential. These findings highlight a dual reality: while organoids offer a clear temporal advantage over traditional *in vivo* models, making them a faster and more scalable option for precision oncology (Bose et al., 2021).The current time frame of four to eight weeks for standard HTO workflows can still limit their utility for frontline therapy selection. Ongoing innovations, such as automated platforms, circulating tumor cell-derived organoids, and real-time imaging, aim to further shorten this timeline, offering both a distinctive advantage and a current limitation in aligning with clinical decision windows (Foo et al., 2022). For instance, droplet microfluidics-based MOS demonstrate that viable, predictive 3D patient-derived microtissues can be generated and assayed on dramatically shortened timelines, offering a concrete route to compress establishment below the multi-week timescale of traditional protocols (Wang et al., 2022). Next-generation platforms may further reduce this window to less than one week through automation and parallelized culture, making perioperative organoid-guided therapy a realistic goal.

At the materials level, replacing Matrigel with chemically defined, synthetic hydrogels (e.g., PEG-derived or other engineered matrices) will reduce batch variability and permit precise tuning of stiffness, ligand presentation, and degradability (Mulero-Russe and García, 2024). Future developments in stimuli-responsive and bio-orthogonal hydrogels could allow real-time modulation of the TME within HTOs, providing a powerful tool for studying therapy-induced adaptation.

Finally, to convert HTOs from static readouts into continuously informative clinical biosensors, they must be linked to longitudinal multi-omics pipelines and modern computational frameworks. Advances in deep-learning-based multi-omics integration and causal-inference methods in oncology provide the analytic foundation to interpret time-series organoid readouts and to suggest causally informed therapeutic adaptations across a patient’s clinical course (Baião et al., 2025; Lan et al., 2024). In the long term, embedding organoids within adaptive AI systems that update predictions as new omics and clinical data accumulate could enable real-time therapy optimization, shifting HTOs from passive testbeds to active decision-making companions in oncology.

### Persisting challenges in HTO application

The application of HTOs in precision oncology is tempered by persistent biological and operational constraints that demand urgent resolution. Foremost among the challenges is the incomplete recapitulation of TME. Although immune co-cultures with T cells, NK cells, or dendritic cells represent tangible progress, they still fail to capture systemic immunodynamics. For instance, neutrophil extracellular trap (NET)-mediated metastasis and the maturation of tertiary lymphoid structures (TLS) remain largely unmodeled, limiting the predictive accuracy of immunotherapy testing (Bagaev et al., 2021; Szczerba et al., 2019). Similarly, immunosuppressive subsets such as regulatory T cells and myeloid-derived suppressor cells are rarely incorporated, despite their critical role in shaping therapeutic resistance (Dwivedi et al., 2022). Future models could address these gaps by incorporating hematopoietic progenitors or engineered immune niches that allow *de novo* differentiation of suppressive and effector subsets, offering a more balanced and clinically relevant immune landscape.

Vascularization also remains an underprioritized microanatomical prerequisite. Perfusable microvascular networks are not merely conduits for nutrient and oxygen delivery but central organizers of metastatic niches, regulators of hypoxic gradients, and mediators of drug penetration heterogeneity. Recent advances in organoids-on-a-chip platforms and endothelial co-culture have demonstrated partial success in creating perfused HTOs, yet scalable and reproducible vascularized systems remain elusive (Li et al., 2024; Park et al., 2025; Zhou et al., 2025). Looking forward, integration of pericyte-stabilized capillaries and lymphatic endothelium, combined with dynamic flow conditions, may yield vascularized organoid models that can support long-term culture and enable quantitative assessment of drug delivery and metastatic spread.

Equally concerning is the oversimplification of CAF heterogeneity. Current co-cultures often lump CAFs into a uniform category, thereby neglecting antigen-presenting CAFs (apCAFs) that directly modulate T-cell activity, inflammatory CAFs (iCAFs) that orchestrate cytokine signaling, and myofibroblastic CAFs (myCAFs) that drive desmoplastic stiffening and invasion dynamics. Meanwhile, the absence of biomechanical force transduction in current models critically undermines the ability of HTOs to model tumor invasion and therapy resistance (Masuda, 2025; Ouni et al., 2025; Wang et al., 2025). Future directions include engineering ECM scaffolds with tunable stiffness and applying mechanical stretch or compression through bioreactors, thereby allowing HTOs to capture the biomechanical regulation of CAF subtypes and their contribution to invasion and therapy resistance.

### Ethical considerations in organoid research

Alongside biological and technical challenges, ethical issues surrounding tumor organoid research are emerging as pivotal and warrant closer scrutiny (Schäffers et al., 2025). To address these concerns, robust governance structures are being developed around organoid biobanks, which involve the long-term storage and controlled sharing of highly personalized samples (Lensink et al., 2021; Perrone and Zilbauer, 2021; Sinha et al., 2025). In China, researchers have drafted standardized operating procedures to regulate sample acquisition, storage, and researcher access, with ethical committee approvals and informed consent as prerequisites (Yang et al., 2024). Globally, scholars emphasize donors’ authentic autonomy and caution against overly broad or blanket consent, which may undermine donors’ capacity for real choice (Lewis and Holm, 2022). Moreover, decentralized biobank models, such as blockchain-enabled platforms, have been proposed to enhance transparency, patient control, and equitable governance (Mollaki, 2021).

Central to these governance efforts is the critical issue of informed consent, especially given the expansive potential uses of organoids in drug screening, genetic analysis, and future commercialization. Traditional one-time, broad consent models may not adequate for respecting donor autonomy in this context (de Jongh et al., 2022). International empirical studies on biobanking show that despite limited public understanding of complex data sharing and privacy issues, potential donors frequently desire greater transparency, control over future uses of samples, and options beyond one-time blanket consent. This evidence strongly supports tiered, dynamic, or governance-based consent models (Kataoka et al., 2024; MacDuffie et al., 2023). Consequently, ethicists advocate for frameworks such as “consent-for-governance” or “dynamic consent”, which emphasize ongoing communication, the possibility of re-consent or withdrawal, and shared oversight over future organoid uses (Boers and Bredenoord, 2018). These discussions are reflected in global normative instruments like the ISSCR Guidelines for Stem Cell Research and Clinical Translation, which call for robust consent procedures, transparency, and governance mechanisms for stem-cell–derived models, including organoids (Lovell-Badge et al., 2021).

Beyond consent, the unique biological nature of organoids introduces significant privacy and protection risks that must be mitigated. Tumor organoids retain the full genomic and phenotypic features of donors, raising risks around re-identification, genetic discrimination, and privacy breaches (Qu et al., 2021). The broader governance structures in China (e.g., Regulations on Human Genetic Resources) offer foundational guidance for human biobank ethics (Shi et al., 2020). Internationally, scholars call for specific regulatory frameworks, public engagement, and continuous monitoring to responsibly govern organoid biobanking (Mollaki, 2021). In conclusion, integrating these ethical issues with technological advancements is of utmost importance for establishing tumor tissue as a scientifically reliable and socially responsible tool in precision oncology.

## Data Availability

Not applicable.

## Abbreviations

AI: artificial intelligence
apCAFs: antigen-presenting CAFs
ASC: adult stem cell
AV-Scaf: acoustic virtual 3D scaffold
BE: barrett’s esophagus
BME: basement membrane extract
CAFs: cancer-associated fibroblasts
CCA: cholangiocarcinoma
CRC: colorectal cancer
CRCLM: colorectal cancer liver metastases
CTCs: circulating tumor cells
DCs: dendritic cells
dECM: decellularized ECM
DMA: methacrylamide dopamine
dMMR: mismatch repair-deficient
Eba-PDOs: embedded bioprinting-enabled arrayed PDOS
ECM: extracellular matrix
EGF: epidermal growth factor
EHS: engelbreth-holm-swarm
ESC: embryonic stem cell
FPCO: fused pancreatic cancer organoid
GBM: glioblastoma
GEMMs: genetically engineered mouse models
HAMA: hyaluronic acid methacrylate
HCCs: hepatocellular carcinomas
HELP: hyaluronan elastin-like protein
HGSOC: high-grade serous ovarian cancer
hiHeps: human induced hepatocytes
HSLCI: high-speed live cells interference imaging
HTOs: human tumor organoids
HUEVCs: human umbilical vein endothelial cells
iCAFs: inflammatory CAFs
IND: investigational new drug
InSMAR-chip: integration of superhydrophobic microwell array chip
iPSC: induced pluripotent stem cell
IPTOs: individualized patient tumor organoids
ISCs: intestinal stem cells
ITH: intra-tumor heterogeneity
KO: knockout
LARC: locally advanced rectal cancer
MAMs: mitochondrion-associated endoplasmic reticulum membranes
mCRC: metastatic CRC
MHC: major histocompatibility complex
MOS: micro-organospheres
MSCs: mesenchymal stem cells
myCAFs: myofibroblastic CAFs
NET: neutrophil extracellular trap
NFs: normal fibroblasts
NGS: next-generation sequencing
NK: natural killer
PDAC: pancreatic ductal adenocarcinoma
PDGCA: patient-derived gastric cancer assembloid
PDMS: polydimethylsiloxane
PDOs: patient-derived organoids
PDXs: patient-derived xenografts
PGOs: patient-derived glioblastoma organoids
PRCC: papillary renal cell carcinoma
PSCs: pluripotent stem cells
QPOP: quadratic phenotypic optimization platform
SA: sodium alginate
saRNAs: small activating rnas
shRNAs: short-hairpin rnas
SMARchip: superhydrophobic micropillar array chips
TA: transit-amplifying
TAM: tumor-associated macrophage
TILs: tumor-infiltrating lymphocytes
TLS: tertiary lymphoid structures
TME: tumor microenvironment
XAI: explainable AI
2D: two-dimensional
3D: three-dimensional
3D-ACS: 3D culture system with a core-shell structure
5-FU: 5-fluorouracil
